# MRI-negative myelitis, especially after COVID-19: a case report and literature review

**DOI:** 10.3389/fimmu.2025.1708018

**Published:** 2025-12-10

**Authors:** Mitsuyoshi Tamura, Yohei Yagi, Satoki Hanayama, Satoko Yoshizaki, Kazumoto Shibuya, Hiroki Masuda, Masahiro Mori

**Affiliations:** Department of Neurology, Graduate School of Medicine, Chiba University, Chiba, Japan

**Keywords:** MRI-negative myelitis, COVID-19, somatosensory evoked potentials, immunotherapy, oligoclonal bands

## Abstract

**Background:**

Neurological sequelae of coronavirus disease 2019 (COVID-19) include inflammatory myelopathies. Among these, magnetic resonance imaging (MRI)-negative myelitis— defined as normal spinal cord MRI findings despite compatible clinical features—presents diagnostic and therapeutic challenges.

**Case presentation:**

A 22-year-old Japanese woman developed progressive distal paresthesia, gait disturbance, bladder and rectal dysfunction, and sensory loss approximately three months after COVID-19. Neurological examination presented with pyramidal tract signs and sensory deficits in both lower limbs. Cerebrospinal fluid oligoclonal bands were positive. Brain MRI showed subtle corticospinal tract hyperintensities, whereas spinal MRI findings remained normal throughout the course. Somatosensory-evoked potentials (SEP) demonstrated absent right N20 and bilateral P37 responses, localizing dysfunction to the thoracic cord. Treatment with intravenous methylprednisolone pulse therapy with plasma exchange resulted in marked clinical recovery and SEP normalization, with only mild residual paresthesia at two-year follow-up.

**Discussion:**

The present case illustrates the clinical utility of SEPs for monitoring disease activity and establishing objective criteria for treatment escalation in post-COVID-19 MRI-negative myelitis. Although MRI-negative myelitis can be observed in myelin oligodendrocyte glycoprotein antibody-associated disease (MOGAD), lupus myelitis, and glial fibrillary acidic protein (GFAP) astrocytopathy, post-COVID-19 myelitis lacks specific biomarkers, complicating both diagnosis and treatment. A review of 20 reported cases of post-COVID-19 MRI-negative myelitis revealed a mean age of 54.4 years, a male-to-female ratio of 3:2, frequent bladder and rectal disturbances and paresis (85% each), high severity (63.2%), a median infection-to-neurological interval of 28 days, oligoclonal bands in 25% (4/16), multiple immunotherapies in 66.7%, and marked improvement or recovery in 66.7%.

**Conclusion:**

In post-COVID-19 MRI-negative myelitis, SEPs offer critical diagnostic and prognostic information. Early recognition and timely escalation of combination immunotherapy may optimize neurological outcomes.

## Introduction

Neurological complications after coronavirus disease 2019 (COVID-19) encompass a spectrum of inflammatory disorders. Magnetic resonance imaging (MRI)-negative myelitis, defined as a normal spinal cord MRI despite a compatible clinical syndrome, has been increasingly reported in this population (1, 2). The absence of abnormalities on MRI can hinder the diagnosis of myelitis and often delay the initiation of immunotherapy. Despite these clinical challenges, its underlying pathophysiology remains poorly understood.

Herein, we report the case of a 22-year-old Japanese woman who developed MRI-negative myelitis following COVID-19. The diagnosis and clinical course were evaluated using somatosensory evoked potentials (SEPs), and the patient was successfully treated with combined immunotherapy.

## Case presentation

**Figure 1:**
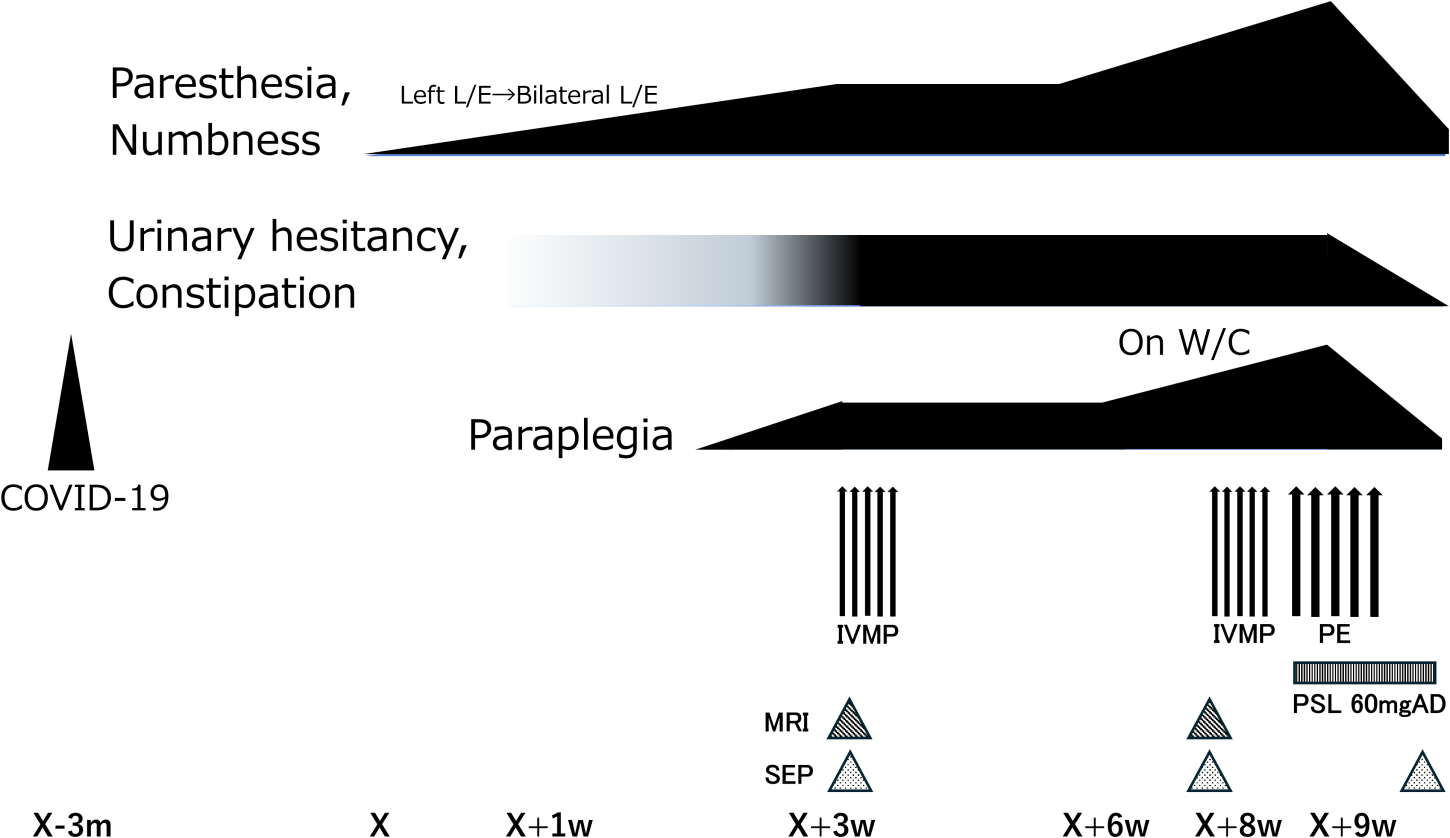
Clinical course table of the present patient. Three months after COVID-19, the patient developed paresthesia and numbness in the lower extremities, followed by progressive onset of urinary hesitancy, constipation, and paraplegia over a three-week period. Following intravenous methylprednisolone pulse therapy (IVMP), symptoms plateaued but did not improve, and three weeks after IVMP, symptoms worsened again. Although repeat MRI showed no apparent lesions, worsening findings on somatosensory evoked potentials (SEP) prompted additional immunotherapy. As gait disturbance requiring a wheelchair persisted despite IVMP, plasma exchange (PE) was performed five times. The treatment was effective, and by the completion of the fifth PE session, all symptoms had markedly improved.

The patient was a 22-year-old Japanese woman with no family history of neurological disease. Five months prior to admission, she contracted COVID-19, as confirmed by antigen testing. During the acute phase of COVID-19, she presented with high fever, severe fatigue, and respiratory symptoms, resulting two weeks of work disability. There were no subsequent infections, antecedent trauma, or vaccination between the COVID-19 infection and neurological symptom onset. Three months after infection, she noticed numbness in her distal lower extremities. In the following weeks, her symptoms progressed to include gait disturbances, urinary hesitancy, and constipation (**Figure 1**). Three weeks after the neurological onset, the patient visited our hospital.

Physical examination on admission revealed diffuse weakness in the lower extremities, which was most pronounced bilaterally in the iliopsoas muscles (Medical Research Council (MRC) grade: right 2/5, left 3/5). Muscle tone increased in the right lower extremity, and deep tendon reflexes were hyperactive in both lower extremities, with bilateral positive Babinski and Chaddock reflexes. Ankle clonus was observed with three beats on the right and two beats on the left. The presence of Lhermitte’s sign was also observed. Pain sensation was reduced below the tenth thoracal (T10). Heel-to-knee testing revealed bilateral dysmetria, which improved with visual guidance. The vibration sensation in the lower extremities and proprioception of the toes was mildly impaired. The patient also exhibited delayed micturition and constipation.

Although a thoracic spinal cord lesion was suspected initially, laboratory and neuroimaging findings were largely unremarkable except for positive cerebrospinal fluid (CSF) oligoclonal bands (OCBs) (type II). Blood test results for vitamins B1, B6, B12, folate, and very-long-chain fatty acids were within the normal ranges. Serological tests for infections, including *Treponema pallidum* antibody, human T-lymphotropic virus type 1 and 2 antibodies, herpes simplex virus, varicella-zoster virus, Epstein–Barr virus, and cytomegalovirus, were negative. Immunological markers, including immunoglobulin G, immunoglobulin A, immunoglobulin M, complement components, and lupus anticoagulants, were within the normal limits. Serum antibodies, including anti-aquaporin-4 (enzyme-linked immunosorbent assay), anti-myelin oligodendrocyte glycoprotein (MOG; live cell-based assay), anti-nuclear, anti-double-stranded DNA, anti-SS-A (Ro), and anti-SS-B (La), were all negative. CSF analysis revealed a normal cell count (1/μL), protein level (24 mg/dL), interleukin-6 (4.2 pg/mL), and immunoglobulin G index (0.49). The cerebrospinal fluid was negative for anti-glial fibrillary acidic protein (GFAP) antibodies.

**Figure 2:**
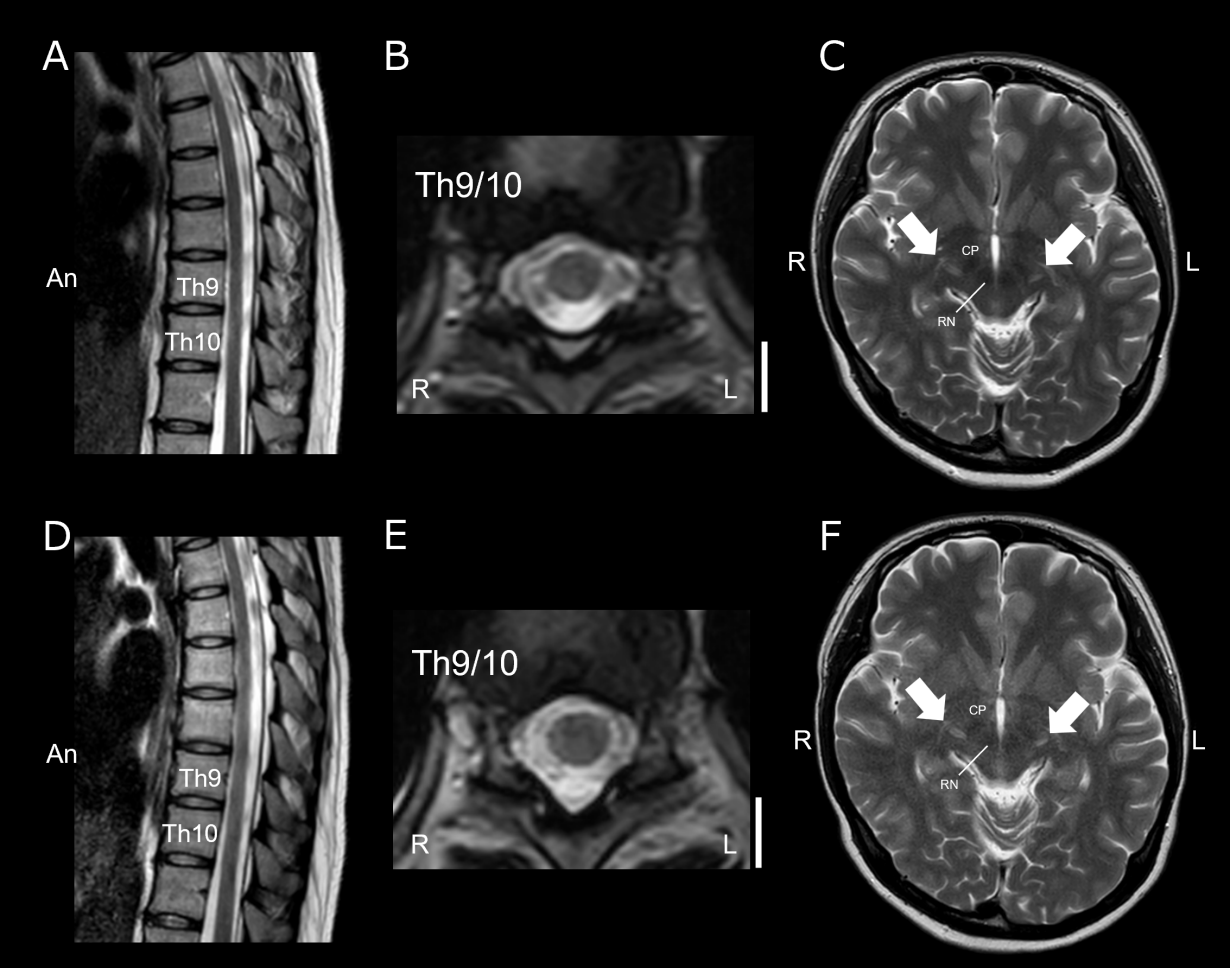
Spinal cord and brain MRI images at the initial hospitalization and one month later. At the initial hospitalization, sagittal T2-weighted images (T2WI) of the thoracic spinal cord **(A)** showed no abnormal lesions. Although clinical findings suggested lesions around the Th9/10 level, axial T2WI showed no intramedullary abnormal signals **(B)** Brain MRI revealed mild hyperintense changes in the bilateral pyramidal tracts on T2WI (**C**, white arrows). Following intravenous methylprednisolone pulse therapy, spinal cord MRI one month later also showed no intramedullary abnormal signals [**(D)** sagittal T2WI; **(E)** axial T2WI at the Th9/10 level]. Brain MRI showing mild hyperintense changes in the bilateral pyramidal tracts on T2WI, similar to the initial findings (**F**, white arrows). White bars correspond to 10 mm. R, right; L, left; An, anterior side; CP, Cerebral peduncle; RN, Red nucleus.

**Figure 3:**
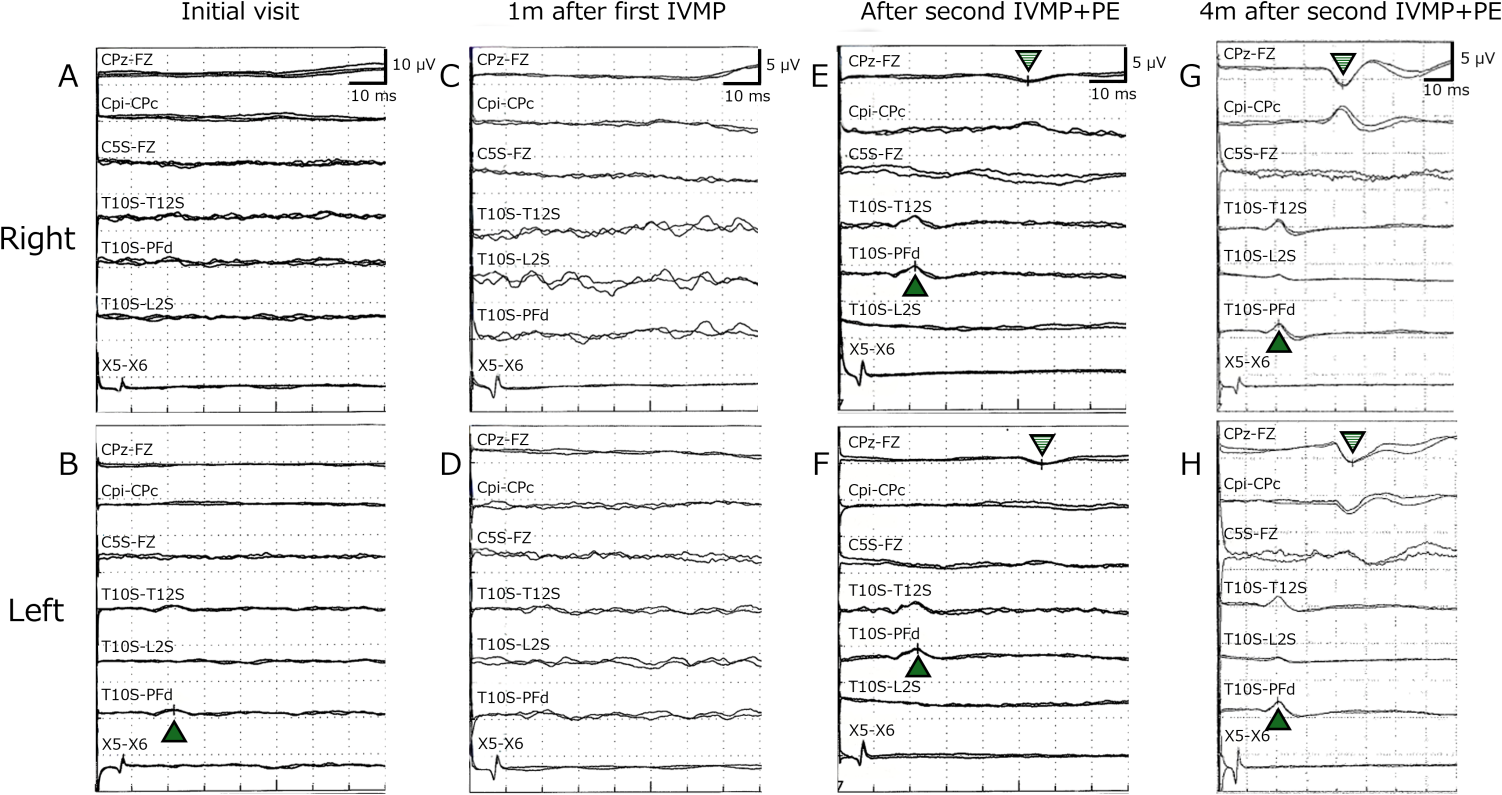
Somatosensory evoked potentials (SEPs) at the initial visit **(A, B)**, one month later after the first intravenous methylprednisolone pulse therapy (IVMP) **(C, D)**, after the second IVMP and plasma exchange (second therapy) **(E, F)**, and four months after the second therapy **(G, H)**. Black arrows indicate N20, and striped arrows indicate P37. At the initial hospitalization, right N20 and bilateral P37 were absent **(A, B)**. On re-examination one month later (after the first IVMP), no waveforms were elicited proximal to the popliteal fossa **(C, D)**. After the second therapy, bilateral N20 and P37 reappeared, with slightly prolonged N20 latencies and markedly prolonged P37 latencies (right N20: 21.5 ms, P37: 52.8 ms; left N20: 22.2 ms, P37: 56.4 ms) **(E, F)**. At four months after the second therapy, N20 and P37 latencies had improved (right N20: 21.0 ms, P37: 43.1 ms; left N20: 20.4 ms, P37: 45.7 ms) **(G, H)**.

MRI of the cervical and thoracic spinal cord revealed no abnormalities (**Figures 2A, B**). Contrast-enhanced MRI of the spinal cord also revealed no abnormal findings. Brain MRI revealed mildly increased signal intensities along the corticospinal tract, which were unlikely to represent plaques, with no other apparent abnormalities (**Figure 2C**). However, SEP studies revealed the absence of the right N20 component and bilateral absence of P37 responses, indicating significant dysfunction of the somatosensory pathway (**Figures 3A, B**).

Based on clinical symptoms, subacute progression, positive OCB in the CSF, and SEP findings, spinal cord damage at the thoracic level caused by an inflammatory condition was suspected. High-dose intravenous methylprednisolone (IVMP: 1,000 mg/day) was administered for five consecutive days; however, the therapeutic response was limited. The patient’s functional status deteriorated, requiring wheelchair assistance one month after the initial treatment. Follow-up spinal cord MRI showed no abnormalities (**Figures 2D, E**); however, re-examination of the SEP confirmed the persistent absence of both N20 and P37 (**Figures 3C, D**), suggesting ongoing somatosensory pathway dysfunction. Brain MRI again revealed hyperintensities along the corticospinal tract (**Figure 2F**). Given the inadequate response to the initial corticosteroid therapy, a second treatment course was initiated a month after the first course, which combined high-dose methylprednisolone with therapeutic plasma exchange. Plasma exchange was selected as additional therapy based on the following considerations: the acute clinical course and positive OCBs suggested autoimmune involvement; the patient was already non-ambulatory and required wheelchair despite receiving IVMP therapy, warranting aggressive treatment intensification; and the target autoantibody remained unidentified. Following combined treatment, the patient demonstrated gradual clinical improvement. Numbness and muscle weakness began improving after the second plasma exchange session, indicating treatment efficacy. Three weeks after initiating retreatment (after five sessions of plasma exchange), the patient regained independent ambulation. Post-treatment SEP studies revealed the reappearance of both N20 and P37 (**Figures 3E–H**), which correlated with clinical recovery. Over two years of follow-up, the patient has remained stable, with only mild residual distal paresthesia.

## Discussion

Our findings from this case reveal four clinically significant takeaways regarding post-COVID-19 myelitis: (1) myelitis can occur even when spinal cord MRI findings are normal, (2) SEPs provide decisive diagnostic and monitoring value in MRI-negative myelitis, (3) COVID-19 may act as a preceding infectious trigger, and (4) escalation-ready immunotherapy can be effective when the initial steroid response is suboptimal.

### MRI-negative myelitis

We identified articles to be included in this narrative review by entering the following search terms into PubMed, Web of Science, and Google Scholar: MRI-negative myelitis and MRI negative myelitis. The clinical features and laboratory findings of patients with MRI-negative myelitis, excluding post-COVID-19 cases reported in the literature, are summarized in **Table 1**. Particularly in MOGAD cases with recurrent relapses, entries in the table were limited to instances that fulfilled the following conditions: spinal cord MRI was performed when myelitis symptoms were present, and MRI-negativity was confirmed at the time. Cases in which a spinal cord lesion was identified on MRI at any point during the clinical course, and cases of multiple sclerosis were excluded. MRI-negative myelitis is a rare clinical entity characterized by symptoms indicative of spinal cord inflammation, despite normal spinal MRI findings. This phenomenon has been recognized in specific contexts, including MOG antibody-associated disease (MOGAD), GFAP astrocytopathy, Sjögren’s syndrome, and lupus myelitis (3–7). When considering the mechanism of MRI-negative myelitis associated with COVID-19, examining the pathogenesis of human T-cell leukemia virus type 1 (HTLV-1)-associated myelopathy/tropical spastic paraparesis (HAM/TSP) –one of the diseases causing myelopathy related to viruses– may provide insight. HAM/TSP is a chronic inflammatory disorder of the spinal cord caused by HTLV-1. The virus primarily infects CD4^+^; T cells, and viral proteins such as Tax and HBZ induce persistent activation and cytokine production (8). In the spinal cord, HTLV-1–infected T cells and HTLV-1–specific CD8^+^; cytotoxic T lymphocytes infiltrate and release proinflammatory cytokines including IFN-γ and TNF-α, leading to blood–brain barrier disruption and secondary demyelination and axonal loss (9). Thus, the pathogenesis is driven mainly by immune-mediated mechanisms rather than direct viral cytotoxicity. In COVID-19-associated MRI-negative myelitis, COVID-19 may contribute by initiating virus-specific immune activation that progressively damages the spinal cord. Previous reports have included cases where it took 5 to 6 months for myelitis to develop after COVID-19 infection (**Table 2**). While it cannot be ruled out that COVID-19 infection is not involved in the development of myelitis in our case, the existence of such complex mechanisms may also be prolonging the incubation period.

**Table 1:** Clinical features and laboratory findings of patients with MRI-negative myelitis, excluding post-COVID-19 cases reported in the literature.

Reference	Age	Sex	Main myelitis symptoms	Severity	MRI timing from onset	Spinal cord MRI	Brain MRI	SEPs	CSF	Acute-phase treatment	Outcome
MOGAD											
Kolcava (2022) (3)	26	M	Bl, S	Mo	10d	normal	normal	suspicious	pro 224 mg/dL	IVCS	Excellent
Viswanathan (2020) (6)	34	F	Bl, Pa	Se	onset, 2m	normal	N/A	N/A	Normal	IVCS	Excellent
Yang (2023) (10)	62	F	Bl, Pa	Mo	7d, 33d	normal	normal	normal	pro 66.7 mg/dL	IVCS	Excellent
Macaron (2020) (11)	57	M	Bl, Qu	Mo	2d, 5d, 121d	normal	normal	N/A	pro 75 mg/dL	IVCS	Excellent
Perez (2019) (12)	8	M	Bl, Pa	Mi	N/A	normal	normal	N/A	pro 97 mg/dL	IVCS	Excellent
Hwangbo (2023) (13)	37	F	Bl	Mi	N/A	normal	normal	normal	pro 48 mg/dL	IVCS	Excellent
Sechi (2021) (14)	35	F	Bl	Mo	2w, 4w	normal	normal	N/A	cell 48/μL, pro 100 mg/dL	IVCS	Excellent
Sechi (2021) (14)	46	M	Mu, Bl	Mo	N/A	normal	optic nerve swelling and enhancement	N/A	cell 161/μL, pro 52 mg/dL	IVCS	Excellent
Sechi (2021) (14)	80	M	Bl, Pa	Mo	N/A	normal	N/A	suspicious	N/A	oral steroid	Excellent
Ortiz-Guerrero (2025) (15)	47	F	S, Pa, Bl	Mi	10d, 2m, 1y	normal	non-specific	N/A	cell 23/μL, pro 48 mg/dL	IVCS	Excellent
Friedman-Korn (2025) (16)	31	F	S, Bl	Mi	onset, 5m	normal	two T2 hyperintense lesions	N/A	normal	IVCS	Excellent
Friedman-Korn (2025) (16)	29	F	S, Mu, Bl	Mi	7d, 3m	normal	N/A	N/A	N/A	IVCS	Excellent
Goto (2021) (31)	35	F	S, Pa, Bl	Mo	N/A	normal	normal	N/A	cell 19/μL, pro 56 mg/dL	IVCS	Excellent
Baddam (2025) (17)	64	M	S, Mu, Bl	Se	OP	normal	non-specific	N/A	normal	IVCS	Improved
GFAP-A											
Goyne (2023) (7)	50s	M	S, Pa, Bl	Se	onset, 11d, 2w	11d: long lesion	linear periventricular radial enhancement	N/A	pleocytosis, HP	IVCS, PE	Excellent
Lupus-M											
Das (2022) (21)	21	F	S, Mu, A	N/A	OP, 7d, 6w, 3m	normal	unidentified bright object	N/A	pleocytosis, HP	steroid, CP, MMF	Excellent
Das (2022) (21)	26	F	S, Mu, A	N/A	OP, 7d, 6w, 3m	normal	normal	N/A	HP	steroid, CP, Rituximab	Excellent
Das (2022) (21)	26	F	Mu, A	N/A	OP, 7d, 6w, 3m	normal	normal	N/A	HP	steroid, CP, MMF	Excellent
Das (2022) (21)	35	F	Mu, A	N/A	OP, 7d, 6w, 3m	normal	normal	N/A	HP	steroid, PE, CP	Excellent
Das (2022) (21)	20	F	Mu, A	N/A	OP, 7d, 6w, 3m	normal	normal	N/A	pleocytosis, HP	steroid	Excellent
Das (2022) (21)	24	F	S, Mu, A	N/A	OP, 7d, 6w, 3m	normal	normal	N/A	pleocytosis, HP	steroid, CP, MMF	Excellent
Das (2022) (21)	29	M	Mu, A	N/A	OP, 7d, 6w, 3m	normal	normal	N/A	HP	steroid, CP, MMF	Excellent
Das (2022) (21)	45	F	Mu, A	N/A	OP, 7d, 6w, 3m	normal	unidentified bright object	N/A	pleocytosis, HP	steroid, CP, MMF	Excellent
Grover (2025) (22)	27	F	S, Pa, Bl	Se	OP, 6w	normal	normal	N/A	pro 90 mg/dL	steroid, MMF	Excellent
Monahan (2020) (4)	42	M	S, Pa, Bl	Mo	OP (10d)	normal	N/A	normal	normal	steroid, CP	Improved
Monahan (2020) (4)	36	F	S	Mi	OP	normal	N/A	normal	normal	steroid, CP	Improved
Monahan (2020) (4)	26	F	S, Pa, Bl	Mo	OP, 12m	normal	N/A	N/A	normal	steroid, CP	Improved
Monahan (2020) (4)	23	F	S, Mu	Mi	OP (1w), 2w, 4m	normal	N/A	normal	normal	Steroid, CP	Improved

MOGAD: myelin oligodendrocyte glycoprotein antibody-associated disease; GFAP-A: glial fibrillary acidic protein astrocytopathy; lupus-M: lupus myelitis; Bl: bladder-rectal disorder; Pa: paralysis; Qu: quadriplegia; DS: deep sensation impairment; S: sensory impairment; Mu: muscle weakness; A: autoimmune disorder; Mo: moderate; Se: severe; Mi: mild; N/A: not available; d: days; w: weeks; m: months; OP: on presentation; OCB: oligoclonal bands; MBP: myelin basic protein; pro: protein; cell: cell counts; HP: high level protein; IgG: immunoglobulin G; IVCS: Intravenous corticosteroid; PE: plasma exchange; ACT: anticoagulant therapy; CP: cyclophosphamide; MMF: Mycophenolate Mofetil

**Table 2:** Clinical features and laboratory findings of patients with post-COVID-19 MRI-negative myelitis in the literature.

Reference	Age	Sex	Main symptoms	Severity	C19-Neuro interval	Timepoint of MRI acquisition	Spinal cord MRI	Brain MRI	SEPs	CSF abnormal findings	Acute-phase treatment	Outcome
Our case	22	F	Bl, Pa, DS, Lh	Se	3m	1m (OP), 2m	normal	HCT	N20, P37	OCB+	IVCS, PE	Excellent
Okumura (2024) (2)	57	M	Bl, Pa, DS, Lh	Se	6w	OP, after PE, every month	normal	normal	P40	MBP 402.9 pg/mL	IVCS, PE, IVIg	Excellent
Kawama (2023) (1)	45	M	Bl, Qu, DS	Mi	14d	OP	normal	normal	N20	OCB+	IVCS	Excellent
Kawama (2023) (1)	50	M	Bl, Pa, DS	Mi	7d	OP	normal	normal	N30, P38	OCB+	IVCS	Excellent
Kawama (2023) (1)	44	M	Bl, Pa, Lh, DS	Mo	10d	OP, 3m	3m: HLPC	normal	P38	OCB+	IVCS, IVIg	Excellent
Okumura (2024) (24)	59	M	Bl, Pa	Se	5w	OP, 4m	4m: HLPC	4m: HCT	N/A	pro 67 mg/dL	IVCS, PE, IVIg	Improved
Okumura (2024) (24)	59	M	Bl, Pa, DS	Se	6w	OP, 4m	4m: HLPC	4m: HCT	N/A	Normal	IVCS, PE, IVIg	Excellent
Okumura (2024) (24)	65	M	Bl, Pa, DS	Se	6w	OP, 4m	4m: HLPC	HCT	N/A	MBP 327 pg/mL	IVCS, PE, IVIg	Excellent
Okumura (2024) (24)	52	M	Bl, Ta	Se	4w	OP, 4m	4m: HLPC	4m: HCT	N/A	pro 54 mg/dL, MBP 500 pg/mL, OCB+	IVCS, PE, IVIg	Partial
Masaad (2022) (25)	32	F	Bl, Pa, Lh	N/A	N/A	OP, +1w	normal	Partial thrombosis	N/A	Normal	IVCS, ACT	Excellent
Abrams (2021) (23)	69	M	Bl, Pa	Se	2w	N/A	normal	normal	N/A	pro 69.4 mg/dL	IVCS, IVIg	No response
Abrams (2021) (23)	64	M	Bl, Pa, Lh	Mo	3-4m	OP	normal	normal	N/A	N/A	IVCS	Partial
Abrams (2021) (23)	64	F	Pa	Mo	5-6m	5-6m (OP)	normal	normal	N/A	N/A	N/A	N/A
Abrams (2021) (23)	58	F	Pa	Mo	3w	3w (OP)	normal	normal	N/A	N/A	IVCS	Partial
Abrams (2021) (23)	63	M	Pa	Mi	2m	2m (OP)	normal	hemangioma	N/A	N/A	N/A	N/A
Memon (2021) (26)	60	F	Bl, Pa, Lh	Se	2m	1m (OP), 4m	4m: HLPC	3m: HCT	N/A	cell 20/μL, pro 81.6 mg/dL	IVCS, PE, ACT	Excellent
Metya (2021) (28)	54	F	Bl, Pa	Se	12d	>3w (OP)	normal	normal	N/A	Pleocytosis	IVCS, IVIg	Partial
Giorgianni (2020) (27)	22	F	Bl, Qu	Se	15d	3d (OP)	normal	normal	N/A	pro 53 mg/dL	therapy for COVID-19	Improved
Zachariadis (2020) (29)	63	M	Bl, Pa	Se	12d	4d (OP), 11d	normal	normal	N/A	cell 16/μL, pro 57.3 mg/dL	IVCS, IVIg	Partial
Zukic S (2022) (30)	57	F	Bl, Pa	Se	2m	OP	normal	normal	N/A	elevated IgG	IVCS	Excellent
Belluci (2024) (32)	50	M	Bl, Pa	Se	1d	3d (OP)	normal	N/A	N/A	normal	lopinavir/ritonavir, dexamethasone, IVIg	Excellent

Bl, bladder-rectal disorder; C19-Neuro interval, Interval between COVID-19 and neurological onsets; Pa, paralysis; Qu, quadriplegia; DS, deep sensation impairment; Mo, moderate; Se, severe (unable to walk by his/her own); Mi, mild; N/A, not available; d, days; w, weeks; m, months; OP, on presentation; HLPC, hyperintensity involving the lateral/posterior column; HCT, hyperintensity along the corticospinal tracts; OCB, oligoclonal bands; MBP, myelin basic protein; pro, protein; cell, cell counts; IgG, immunoglobulin G; IVCS, Intravenous corticosteroid; IVIg, Intravenous immunoglobulin; PE, plasma exchange; ACT, anticoagulant therapy.

MOGAD cases typically show visible longitudinally extensive transverse myelitis or central gray matter–predominant cord lesions on early MRI, which are sometimes accompanied by optic neuritis. A serological marker (MOG IgG) has also been established (3, 6, 10). Sustained MRI negativity is uncommon, and relapse is more characteristic than in COVID-19-related myelitis. Steroid treatment alone was highly effective (3, 6, 10–17). Given that 20–40% of patients with MOGAD are reported to have moderate to severe disability (18–20), steroid responsiveness in MRI-negative cases is considered relatively favorable. The proposed mechanisms underlying MRI-negative myelitis in MOGAD include the limited sensitivity of MRI, lesions confined to the gray matter, small lesion size, and susceptibility of MRI to motion artifacts (16). Cases of GFAP astrocytopathy frequently demonstrate radial perivascular enhancement in the brain and longitudinal cord lesions and are supported by GFAP-IgG (often CSF-restricted). Persistent MRI-negative spinal disease is atypical (7). Lupus myelitis often shows early MRI positivity with extensive transverse myelitis, potentially influenced by vascular or inflammatory mechanisms (including antiphospholipid antibodies) (21). Management frequently involves a combination of high-dose steroids with anticoagulation and/or immunosuppressive therapy. Definitive conclusions regarding treatment responsiveness in lupus myelitis have not been established, but at least, MRI-negative cases demonstrate favorable treatment responsiveness (4, 21, 22).

Beyond these conditions, persistence of a normal spinal cord MRI throughout the disease course is rare (5). In this regard, accumulating reports of MRI-negative myelitis following COVID-19 raise the possibility of a distinct post-infectious neuroimmunological syndrome, with pathophysiology different from myelitis associated with other viral infections.

### Post-COVID-19 MRI-negative myelitis

In the present case, the acute onset of myelopathic symptoms with rapid improvement following immunotherapy was consistent with an inflammatory condition. While a definitive causal relationship with prior COVID-19 infection cannot be established owing to the three-month interval before symptom onset, it is noteworthy that a substantial proportion of previously reported cases of MRI-negative myelitis following COVID-19 also demonstrated a latency exceeding three months. Previously reported cases of MRI-negative myelitis following COVID-19 and their clinical features and laboratory findings are summarized in **Table 2** (1, 2, 23–32). According to the previous reports, the mean age of onset was 54.4 years, with a male-to-female ratio of 3:2. The most frequent presenting symptoms were bladder and rectal disturbances (85%), and paresis (85%). The median interval from COVID-19 onset to neurological symptoms was 28 days (mean 39.2 days). OCB positivity was observed in 4 of 16 cases with documented test results. These reports frequently described a subacute progressive course, clinical signs indicative of thoracic cord involvement, and persistently normal spinal cord MRI findings. Compared with previous reports, our case was notable for the prolonged interval between the patient’s initial SARS-CoV-2 infection and the onset of neurological symptoms, as well as the presence of positive OCBs. Our case demonstrated a longer interval of 3 months, as compared to the previously reported median interval of 28 days. The clinical presentation and course were consistent with those of previously reported cases of post-COVID-19 MRI-negative myelitis.

The proposed mechanisms for post-COVID-19 neurological complications include direct viral invasion, systemic inflammatory responses, and molecular mimicry, leading to autoimmune reactions (1, 2). The reasons why spinal cord symptoms may be evident despite negative MRI findings include not only the limitations of MRI sensitivity or lesion size, as discussed in reports on MOGAD (16), but also the possibility that microglial activation, which can reflect disease activity in certain forms of myelitis, does not necessarily manifest as an MRI abnormality. A similar mechanism may be involved in post-COVID-19 myelitis (33). After SARS-CoV-2 infection, immune dysregulation and cytokine release (IL-6, TNF-α, IL-1β) can cause microglial and astrocytic activation and endothelial injury, leading to neuroinflammation without producing the edema or demyelination that generate high T2 signal on MRI (34, 35). In addition, autoimmune reactions triggered by molecular mimicry between viral and neural antigens can disrupt neuronal transmission through antibodies or T-cell responses, causing functional conduction block without structural tissue loss (36). The resulting damage often involves subtle axonal or metabolic changes below the spatial and contrast resolution of conventional MRI sequences. Therefore, MRI negativity arises because the inflammatory and metabolic disturbances are too fine or transient to alter water content or tissue structure sufficiently for MRI detection. Advanced techniques such as diffusion tensor imaging or MR spectroscopy may reveal these invisible microstructural abnormalities.

The temporal relationship and potential causal mechanisms between COVID-19 and delayed-onset myelitis remain under investigation. A previous report described a case of post-COVID-19 myelitis that presented with acute myelopathic symptoms, in which the diagnosis was established on the basis of responsiveness to immunotherapy and the presence of anti-ACE2 antibodies, despite cerebrospinal fluid analysis revealing only mild protein elevation (32). Treatment approaches for MRI-negative myelitis after COVID-19 are not yet well established. Although IVMP is the standard first-line therapy, combination with therapeutic plasma exchange may provide additional benefit, particularly in steroid-refractory cases (1, 26).

Previous studies have described a T2 hyperintensity along the corticospinal tract in the brain MRI of patients with post-COVID-19 MRI-negative myelitis—a phenomenon termed the “grasshopper sign” (24). This subtle radiological feature was observed in our patient, further supporting the proposed clinical entity. This finding may serve as a supportive diagnostic clue in patients with suspected post-COVID-19 spinal cord involvement, particularly when the spinal cord MRI findings remain normal.

Compared with COVID-19–unrelated cases, COVID-19–related MRI-negative myelitis is characterized by (i) MRI-negative spinal cord imaging findings at early stages, (ii) reliance on CSF testing and neurophysiological assessment for early diagnostic confirmation but absence of definitive biomarkers, and (iii) generally favorable responsiveness to combined immunotherapies, although partial responses are seen in some cases.

### Use of SEPs in MRI-negative myelitis

The present case is noteworthy because SEPs were employed to assess disease progression, thereby providing an objective rationale for therapeutic intensification of post-COVID-19 MRI-negative myelitis. SEPs provide an objective assessment of the integrity of the somatosensory pathway and can detect subclinical dysfunction when conventional MRI is unrevealing (37). They serve as valuable diagnostic tools and biomarkers for monitoring treatment responses in patients with suspected spinal cord pathology but normal neuroimaging findings. Although clinical knowledge regarding post-COVID-19 MRI-negative myelitis is expanding, few reports have demonstrated objective treatment responses using electrophysiological markers (1). In our case, the reappearance of the N20 and P37 components in the SEP following immunotherapy provided compelling neurophysiological evidence of recovery, aligning well with the observed clinical improvement. These findings highlight the diagnostic and monitoring values of SEPs in this rare clinical context. In addition, SEP is pivotal in revealing subclinical dysfunction and provides objective justification for initiating an intensive therapeutic approach, underscoring its essential role in both diagnosis and management.

The association between COVID-19 and myelitis in our case remains unclear, partly because of the absence of direct virological evidence. While combined immunotherapy was considered effective in our case, the possibility that the initial IVMP treatment exerted a delayed therapeutic effect cannot be entirely excluded, potentially confounding the apparent superiority of combination therapy. The mechanisms underlying MRI-negative myelitis, particularly after COVID-19, as well as optimal treatment strategies, require further investigation through systematic studies.

## Conclusion

COVID-19 can cause delayed-onset myelitis, which may present as persistently normal spinal cord MRI findings. SEPs may be valuable for both the diagnosis and monitoring of treatment responses in patients with MRI-negative myelitis.

## Data Availability

The original contributions presented in the study are included in the article/supplementary material. Further inquiries can be directed to the corresponding author.

## References

[1] Kawama K Shimazaki R Sunami Y Miyakoshi N Tobisawa S Shimizu T . Case report: MRI-negative myelitis following COVID-19 with SEP abnormalities: a case series and literature review. Front Neurol. (2023) 14:1275696. doi: 10.3389/fneur.2023.1275696, PMID: 38020593 PMC10643519

[2] Okumura M Sato T Masui M Kokubu T Umehara T Okamoto T . Magnetic resonance imaging/cerebrospinal fluid-negative myelitis following COVID-19 with a dramatic response to early aggressive immunosuppressive therapy. Intern Med. (2024) 63:2199–201. doi: 10.2169/internalmedicine.3588-24, PMID: 38749730 PMC11358746

[3] Kolcava J Rajdova A Vlckova E Stourac P Bednarik J . Relapsing MRI-negative myelitis associated with myelin-oligodendrocyte glycoprotein autoantibodies: a case report. BMC Neurol. (2022) 22:313. doi: 10.1186/s12883-022-02837-5, PMID: 36002821 PMC9400333

[4] Monahan RC Beaart HJL Fronczek R Terwindt GM Beaart-van de Voorde LJJ de Bresser J . Suspected transverse myelitis with normal MRI and CSF findings in a patient with lupus: what to do? A case series and systematic review. Neuropsychiatr Dis Treat. (2020) 16:3173–86. doi: 10.2147/NDT.S267000, PMID: 33376333 PMC7764958

[5] Cacciaguerra L Sechi E Rocca MA Filippi M Pittock SJ Flanagan EP . Neuroimaging features in inflammatory myelopathies: A review. Front Neurol. (2022) 13:993645. doi: 10.3389/fneur.2022.993645, PMID: 36330423 PMC9623025

[6] Viswanathan LG Kumar S Pramod MN-B . Post-varicella anti-myelin oligodendrocyte glycoprotein antibody-associated magnetic resonance imaging-negative myelitis. Clin Exp Neuroimmunology. (2021) 12:122–3. doi: 10.1111/cen3.12618

[7] Goyne CE Piccioni D Handwerker J Rajagopal A . Radiographic evolution of myelitis in a case of glial fibrillary acidic protein (GFAP) astrocytopathy. BMJ Case Rep. (2023) 16:e248921. doi: 10.1136/bcr-2022-248921, PMID: 36898710 PMC10008168

[8] Yamano Y Sato T . Clinical pathophysiology of human T-lymphotropic virus-type 1-associated myelopathy/tropical sp*astic paraparesis*. Front Microbiol. (2012) 3:389. doi: 10.3389/fmicb.2012.00389, PMID: 23162542 PMC3494083

[9] Bangham CRM . The immune control and cell-to-cell spread of human T-lymphotropic virus type 1. J Gen Virol. (2003) 84:3177–89. doi: 10.1099/vir.0.19334-0, PMID: 14645900

[10] Yang J Lee YB Park HM . MRI-negative myelitis associated with MOG-IgG antibody: A case report and literature reviews. eNeurologicalSci. (2023) 33:100481. doi: 10.1016/j.ensci.2023.100481, PMID: 37886214 PMC10598690

[11] Macaron G Ontaneda D . MOG-related disorders: A new cause of imaging-negative myelitis? Mult Scler. (2020) 26:511–5., PMID: 30931813 10.1177/1352458519840746

[12] Pérez CA Garcia-Tarodo S Troxell R . MRI-negative myelitis associated with myelin oligodendrocyte glycoprotein antibody spectrum demyelinating disease. Child Neurol Open. (2019) 6:2329048x19830475. doi: 10.1177/2329048X19830475, PMID: 30800700 PMC6379793

[13] Hwangbo J Oh SI . A case of MRI-negative encephalomyelitis in a patient with long-term stable myelin oligodendrocyte glycoprotein antibody-associated disease. J Clin Neurol. (2023) 19:422–4. doi: 10.3988/jcn.2022.0433, PMID: 37417441 PMC10329926

[14] Sechi E Krecke KN Pittock SJ Dubey D Lopez-Chiriboga AS Kunchok A . Frequency and characteristics of MRI-negative myelitis associated with MOG autoantibodies. Mult Scler. (2021) 27:303–8. doi: 10.1177/1352458520907900, PMID: 32103708 PMC7500857

[15] Ortiz-Guerrero G Mafla D Carter C Patel A . Diagnostic and treatment challenges in MRI-negative myelitis associated with MOG antibody: A case report and literature review. RRNMF Neuromuscular J. (2025) 6:16. doi: 10.17161/rrnmf.v6i1.21721

[16] Friedman-Korn T Rechtman A Zveik O Vaknin-Dembinsky A . Repeated MRI-negative demyelinating attacks linked to MOG-IgG antibodies and silent lesions - Case series and literature review. Clin Neurol Neurosurg. (2025) 257:109111. doi: 10.1016/j.clineuro.2025.109111, PMID: 40834627

[17] Baddam S Patel S Kahlon N Thiriveedi M . Unmasking myelin oligodendrocyte glycoprotein antibody-associated disease (MOGAD): CNS demyelination triggered by TNF-α Inhibition in a patient with ankylosing spondylitis. Eur J Case Rep Intern Med. (2025) 12:005467. doi: 10.12890/2025_005467, PMID: 40502939 PMC12151557

[18] Jarius S Ruprecht K Kleiter I Borisow N Asgari N Pitarokoili K . MOG-IgG in NMO and related disorders: a multicenter study of 50 patients. Part 2: Epidemiology, clinical presentation, radiological and laboratory features, treatment responses, and long-term outcome. J Neuroinflamm. (2016) 13:280. doi: 10.1186/s12974-016-0718-0, PMID: 27793206 PMC5086042

[19] Jurynczyk M Messina S Woodhall MR Raza N Everett R Roca-Fernandez A . Clinical presentation and prognosis in MOG-antibody disease: a UK study. Brain. (2017) 140:3128–38. doi: 10.1093/brain/awx276, PMID: 29136091

[20] Deschamps R Pique J Ayrignac X Collongues N Audoin B Zéphir H . The long-term outcome of MOGAD: An observational national cohort study of 61 patients. Eur J Neurol. (2021) 28:1659–64. doi: 10.1111/ene.14746, PMID: 33528851

[21] Das S Ray BK Chakraborty AP Banerjee A Pandit A Das G . Persistent “MRI-negative” lupus myelitis-disease presentation, immunological profile and outcome. Front Neurol. (2022) 13. doi: 10.3389/fneur.2022.968322, PMID: 36388234 PMC9659815

[22] Grover D Shakya H Panda AK Hazra S Dung AAD Kushwaha S . MRI-negative central nervous system SLE: A unique case report. Ann Indian Acad Neurol. (2025) 28:145–7. doi: 10.4103/aian.aian_654_24, PMID: 39632407 PMC11892959

[23] Abrams RMC Safavi F Tuhrim S Navis A Steinberger J Shin SC . MRI negative myelopathy post mild SARS-CoV-2 infection: vasculopathy or inflammatory myelitis? J Neurovirol. (2021) 27:650–5. doi: 10.1007/s13365-021-00986-w, PMID: 34101085 PMC8186350

[24] Okumura M Sekiguchi K Okamoto T Saika R Maki H Sato W . ‘Grasshopper sign’: the novel imaging of post-COVID-19 myelopathy with delayed longitudinal white matter abnormalities. BMJ Neurol Open. (2024) 6:e000730. doi: 10.1136/bmjno-2024-000730, PMID: 38884066 PMC11177679

[25] Masaad D Youssef S Safadi MF Shehadeh Agha M . MRI-negative myelitis associated with cerebral venous thrombosis after COVID-19 infection. BMJ Case Rep. (2022) 15. doi: 10.1136/bcr-2022-250535, PMID: 36307141 PMC9621158

[26] Memon AB Al-Hader R Patel S Malik S Megally M Steijlen KL . Late-onset rapidly progressive MRI- negative-myelitis after COVID-19 illness. Clin Neurol Neurosurg. (2021) 202:106513. doi: 10.1016/j.clineuro.2021.106513, PMID: 33517162 PMC7825887

[27] Giorgianni A Vinacci G Agosti E Cariddi LP Mauri M Baruzzi F . Transient acute-onset tetraparesis in a COVID-19 patient. Spinal Cord. (2020) 58:1042–4. doi: 10.1038/s41393-020-0493-8, PMID: 32488194 PMC7264484

[28] Metya S Shaw S Mondal S Chakraborty B Das S Roy S . MRI-negative myeloradiculoneuropathy following Covid-19 infection: An index case. Diabetes Metab Syndr. (2021) 15:102305. doi: 10.1016/j.dsx.2021.102305, PMID: 34653903 PMC8492362

[29] Zachariadis A Tulbu A Strambo D Dumoulin A Di Virgilio G . Transverse myelitis related to COVID-19 infection. J Neurol. (2020) 267:3459–61. doi: 10.1007/s00415-020-09997-9, PMID: 32601756 PMC7322383

[30] Zukic S Topcic E Hodzic R Sinanovic O Vidovic M . Spastic paraparesis after SARS-CoV-2 infection without radiological changes. Cureus. (2022) 14:e23054. doi: 10.7759/cureus.23054, PMID: 35419244 PMC8994857

[31] Goto H Suetsugi N . Anti-myelin oligodendrocyte glycoprotein antibody-associated disease presenting MRI-negative myelitis and papilledema. Neurol Clin Neurosci. (2021) 9:502–3. doi: 10.1111/ncn3.12544

[32] Bellucci M Bozzano FM Castellano C Pesce G Beronio A Farshchi AH . Post-SARS-CoV-2 infection and post-vaccine-related neurological complications share clinical features and the same positivity to anti-ACE2 antibodies. Front Immunol. (2024) 15:1398028. doi: 10.3389/fimmu.2024.1398028, PMID: 39148725 PMC11324485

[33] Takewaki D Lin Y Sato W Ono H Nakamura M Araki M . Normal brain imaging accompanies neuroimmunologically justified, autoimmune encephalomyelitis. Neurol Neuroimmunol Neuroinflamm. (2018) 5:e456. doi: 10.1212/NXI.0000000000000456, PMID: 29616233 PMC5880628

[34] Paterson RW Brown RL Benjamin L Nortley R Wiethoff S Bharucha T . The emerging spectrum of COVID-19 neurology: clinical, radiological and laboratory findings. Brain. (2020) 143:3104–20. doi: 10.1093/brain/awaa240, PMID: 32637987 PMC7454352

[35] Iadecola C Anrather J Kamel H . Effects of COVID-19 on the nervous system. Cell. (2020) 183:16–27. doi: 10.1016/j.cell.2020.08.028, PMID: 32882182 PMC7437501

[36] Zanin L Saraceno G Panciani PP Renisi G Signorini L Migliorati K . SARS-CoV-2 can induce brain and spine demyelinating lesions. Acta Neurochir (Wien). (2020) 162:1491–4. doi: 10.1007/s00701-020-04374-x, PMID: 32367205 PMC7197630

[37] Mohammed HJ Hammady MM Abbas FN . A comparison between somatosensory evoked potentials and spine MRI in the diagnosis of non-compressive myelopathy: which is more accurate? Cureus. (2023) 15:e38051. doi: 10.7759/cureus.38051, PMID: 37228549 PMC10207993

